# Voltammetric Sensor Based on Molecularly Imprinted Chitosan-Carbon Nanotubes Decorated with Gold Nanoparticles Nanocomposite Deposited on Boron-Doped Diamond Electrodes for Catechol Detection

**DOI:** 10.3390/ma13030688

**Published:** 2020-02-04

**Authors:** Coral Salvo-Comino, Ilhem Rassas, Sylvain Minot, Francois Bessueille, Madjid Arab, Virginie Chevallier, Maria Luz Rodriguez-Mendez, Abdelhamid Errachid, Nicole Jaffrezic-Renault

**Affiliations:** 1 Institute of Analytical Sciences UMR CNRS-UCBL-ENS 5280, University of Lyon, 69100 Villeurbanne, France; coraldeugena@hotmail.com (C.S.-C.); ilhemras@hotmail.fr (I.R.); sylvain.minot@isa-lyon.fr (S.M.); francois.bessueille@univ-lyon1.fr (F.B.); abdelhamid.errachid@univ-lyon1.fr (A.E.); 2 Group UVASens. Dpt. Inorganic Chemistry, Engineers School, University of Valladolid, 47011 Valladolid, Spain; 3 BioecoUVA Institute, University of Valladolid, 47011 Valladolid, Spain; 4 University of Toulon, AMU, CNRS, IM2NP, CS 60584, CEDEX 9, F-83041 Toulon, France; madjid.arab@im2np.fr (M.A.); chevallier@univ-tln.fr (V.C.)

**Keywords:** molecular imprinted polymer, electrochemical sensor, catechol, chitosan, BDD electrode

## Abstract

Phenolic compounds such as catechol are present in a wide variety of foods and beverages; they are of great importance due to their antioxidant properties. This research presents the development of a sensitive and biocompatible molecular imprinted sensor for the electrochemical detection of catechol, based on natural biopolymer-electroactive nanocomposites. Gold nanoparticle (AuNP)-decorated multiwalled carbon nanotubes (MWCNT) have been encapsulated in a polymeric chitosan (CS) matrix. This chitosan nanocomposite has been used to develop a molecular imprinted polymers (MIP) in the presence of catechol on a boron-doped diamond (BDD) electrode. The structure of the decorated MWCNT has been studied by TEM, whereas the characterization of the sensor surface has been imaged by AFM, demonstrating the satisfactory adsorption of the film and the adequate coverage of the decorated carbon nanotubes on the electrode surface. The electrochemical response of the sensor has been analyzed by cyclic voltammetry (CV) where excellent reproducibility and repeatability to catechol detection in the range of 0 to 1 mM has been found, with a detection limit of 3.7 × 10^−5^ M. Finally, the developed sensor was used to detect catechol in a real wine sample.

## 1. Introduction

Due to the advantages that antioxidants offer to health—protecting cells from free radical damage, among others—it is of special interest to include antioxidants, present in food and beverages, in diets. One class of biological antioxidants are phenols and polyphenols, as they can inhibit the oxidation of lipoproteins that protect from cardiovascular illness and cancer [1,2]. The advantage of using sensors instead of traditional analytical methods such as high-performance liquid chromatography (HPLC) [3], spectrophotometry [4,5], or electrophoresis [6] is due to their simplicity, the possibility of miniaturization, their fast response, and low cost. One of the techniques most employed for their detection in the food industry is based on electrochemical biosensors and sensors. The detection of phenolic compounds is of special importance in the wine industry due to the need to guarantee the organoleptic properties of each kind of wine [7].

The non-enzymatic electrochemical detection of catechol has been improved based on different electroactive layers [8,9], where the thinness of this film can be controlled through different deposition techniques, such as electrodeposition [10], layer by layer [11], Langmuir–Blodgett [12], and spin-coating [13] among others.

The mechanism of MIP (molecular imprinted polymers) is based on the development of a polymeric film, which contains the template molecule that is removed at the end of the deposition and generates specific recognition cavities for the detection molecule [14,15]. The reproducibility, stability and ease of preparation of the MIP sensors suggest a promising technique in the development of electrochemical sensors for the detection of phenols.

It has been demonstrated that the polymeric matrix has to be selected meticulously to ensure proper assembly between the target molecule and the film. The selection of biopolymers has been of special interest due to their biocompatibility in the development of biosensors [15]. The employment of one biocompatible amino-polysaccharide derived from the shells of crustaceans, chitosan (CS) presents some advantages due to its non-toxic activity and excellent permeability; moreover, it has an excellent layer forming ability. However, due to the high degree of crystallinity that the polymer has, conductivity is low, which hinders the redox process [16]. To overcome these drawbacks, the formation of a nanocomposite including nanomaterials with electroactive functionality is of great interest in the development of electrochemical sensors, due to the fact that nanomaterials—such as carbon nanotubes (CNT), metal nanoparticles (MNP), graphene, phthalocyanines metal nanowires, and metal nanorods among others—can improve the electron transfer between the electrode surface and the solution, working as electron mediators [17].

CNT have been used to enhance the performance of polymers due to their mechanical strength, thermal stability, electrical, chemical, and structural properties; however, the main disadvantage, their poor dispersion in polymeric matrix, makes it difficult to modify a polymeric matrix with this nanomaterial [18]. This disadvantage can be addressed by prior surface modification of the CNT. It has been demonstrated that CNT functionalized with polyelectrolytes display homogeneous dispersion and increase conductivity [17]. Moreover, the employment of AuNP in combination with this conductive material increase the electroconductivity and electrocatalytic behavior; concretely, the combination of CNT and AuNP can amplify the electrochemical performance of the sensor [19].

Of all the possible supports available for electrochemical performance, boron-doped diamond electrodes (BDD) have excellent properties when used as supports in electrochemical sensors due to their excellent chemical and mechanical stability [20]. They are also a perfect option for the detection of catechol because they have a large potential window and a low background current; in addition, they are biocompatible, which in combination with a biopolymer film makes possible their use in the biological matrix [21]. We have performed in our previous work [22] the first MIP chitosan film electrodeposited on BDD electrode for the successful detection of catechol. The aim of this research was to combine the electrocatalytic properties of nanomaterials, MWCNT decorated with AuNP, and their encapsulation in the polymeric matrix of CS, to increase electron transfer in the MIP film. The analytical performance of the MIP sensor was determined in buffer and it was applied to the detection of phenol in red wine samples.

## 2. Materials and Methods

### 2.1. Chemicals

Acetic acid (99.8%), chitosan (MW = 45 kDa, degree of acetylation >75.0%), hydrogen tetrachloroaurate III trihydrate (HAuCl_4_·3H_2_O, 99.9%), and *N*-(2-Hydroxyethyl) piperazyne-*N*’-(2-ethanesulfonic acid) (HEPES), ammonia solution, glutaraldehyde solution, pyrocatechol, 2-aminothiophenol (≥99.0%), bisphenol-A (≥99.0%), 2-nitrophenol (98.0%), 4-aminothiophenol (97.0%), and 4-tert-butylcatechol (≥99.0%) were purchased from Sigma Aldrich. Phosphate buffered saline (PBS; pH 7.4, 0.1 M) was prepared with Na_2_HPO_4_, NaH_2_PO_4_ and NaCl salt also obtained from Sigma Aldrich (Darmstadt, Germany). Tri-sodium citrate dihydrate (99.0%) was obtained from Alfa Aesar (Kandel, Germany). Acetone, ethanol (98%), sulfuric acid (95%), hydrogen peroxide (30.0% in water), and 4-nitrophenol (≥99.5%) were purchased from Fluka (München, Germany).

Aqueous solutions were prepared using Milli Q water (resistivity 18.2 MΩ∙cm).

Boron-doped diamond electrodes were provided by NEOCOAT company (La Chaux-de-Fonds, Switzerland). 300 nm thick polycrystalline boron-doped diamond with boron concentration higher than 7000–8000 ppm was grown by MPECVD on a highly doped silicon substrate.

### 2.2. Instruments

The performance of the electrochemical sensors was recorded by cyclic voltammetry (CV) at room temperature using a Voltalab 80 model PGZ 402 analyzer instrument (Hach Lange, France) connected to Voltamaster 4.0 software. The 5 mL electrochemical cell was based on a three electrode system: a platinum plate as an auxiliary electrode (0.19 cm^2^ of active surface), a saturated calomel electrode (SCE) as a reference electrode and a modified boron-doped diamond electrode (BDD) as the working electrode (0.07 cm^2^ of active surface). CV detection of catechol was performed in 0.1M PBS solution, pH 7.4, at a scan rate of 100 mV/s from −800 to 1500 mV. Electrochemical Impedance spectroscopy (EIS) (initial potential E = 0.2 V, highest freq = 100 kHz, lowest freq = 1 Hz) was used to investigate the charge transfer resistance of the film. EIS measurements were performed in 5 mM ferro-ferricyanide in a phosphate buffer saline solution (PBS).

The AFM measurements were carried out using an Agilent 5500 AFM (Agilent Technologies, Palo Alto, CA, USA). Silicon tips with a nominal spring constant of 20 Nm^−1^ were used in tapping mode at a frequency of ~300 kHz.

The morphology and the size distribution of the MWCNT/AuNP composites, as well as their chemical homogeneity were studied using a FEI Tecnai G2 transmission electron microscope operating at 200 kV coupled to a dispersive energy spectrometer (EDS). HREM images were also obtained on the same microscope.

### 2.3. Preparation of MWCNT/AuNP

The elaboration of MWCNT decorated with AuNP required first the preparation of the aqueous gold colloids and carbon nanotubes. Turkevitch et al.’s [23] method modified by Frens [24] was employed to prepare spherical gold nanoparticles in aqueous solution. The multi-walled CNT preparation was based on the catalytic chemical vapor deposition (CCVD) method reported in previous works [25]. Finally, the decoration of the AuNP on the MWCNT was obtained by dispersion of 50 mg of MWCNT in 15 mL of ethanol under ultrasonication for 30 min. Then 0.5 mL of the CNT suspension was added to 5.0 mL of aqueous gold colloid and the mixture was ultrasonicated for 10 min. The mixture was stored at room temperature for a minimum of 3 days prior to use.

### 2.4. Preparation of CS-MWCNT/AuNP

The CS-MWCNT/AuNP solution was based on non-covalent deposition. 5 mg of CNT/AuNP was dispersed in 10 mL of CS solution (5 mg in 10 mL of 1% vol. acetic acid) and the pH solution was adjusted to 9 with hydroxide ammonia solution. Afterwards, the cross-linking reaction was performed by the addition of 7.2 µL of glutaraldehyde (25% vol.) and the suspension was heated for 1 h. Subsequently, the product obtained was centrifuged at 8000 rpm for 20 min and washed with 1% vol. acetic acid.

### 2.5. Preparation of MIP CS-MWCNT/AuNP Nanocomposite Modified BDD Electrode

1.5 mg/mL of CS-MWCNT/AuNP solution was added to a mixture of CS solution of 1.5 mg/mL prepared by dissolving 1.5 mg in 1 mL of HEPES buffer solution at pH 7.4 and commercial acetic acid (7:3 v/v) containing the desired concentration and 0.1M catechol; subsequently the mixture was ultrasonicated during 1 h at room temperature.

The pretreatment of the BDD electrodes (10 × 10 × 10 mm) was based on immersion in acetone for 10 min under ultrasonication and then the electrodes were washed in Milli Q water; after that, BDD electrodes were cleaning in piranha solution (H_2_SO_4_:H_2_O_2_ = 3:1 v/v proportion) for 5 min; finally, they were washed with Milli Q water and ethanol and dried under a nitrogen flow.

The obtained solution was deposited by drop-casting on the pretreated BDD electrode and stored at room temperature for 3 days for complete drying of the film. The template molecule was eluted in 0.1 M KCl solution under stirring during 20 min.

The molecular non-imprinted polymer (NIP) was prepared under the same conditions in the absence of the catechol template.

### 2.6. Wine Sample Pretreatment

To study the detection of catechol and the behavior of the prepared sensor in a complex matrix, a red wine, Cabernet Sauvignon, was purchased from a supermarket. The wine sample was diluted at 10% in PBS 0.1 M. Finally, the standard addition method was employed to detect catechol in the wine sample by CV.

## 3. Results and Discussion

### 3.1. Morphological Characterization of MIP CS-MWCNT/AuNP Nanocomposite

**Figure 1:**
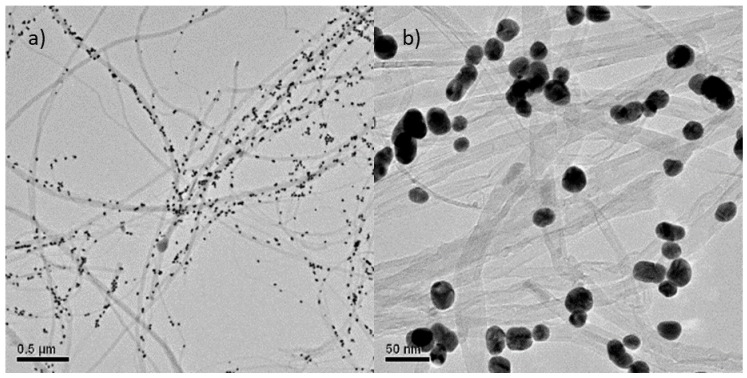
TEM images of multi-walled CNT/AuNP composite in (**a**) 0.5 µm and (**b**) 50 nm scale.

The characterization of the morphology of the MWCNT/AuNP composite was performed by TEM microscopy analyses. Figure 1 shows random dispersion of the nanotubes in two different scales decorated with spherical AuNPs with diameter around 40 nm [26]. The majority of the AuNP deposited onto the MWCNT have been controlled size (40 nm diameter) and shape (spherical); however, as the referee comments, some AuNP aggregations exist wherein several aggregated AuNP appear as an oval structure. The shape of the obtained MWCNT, is that of thin wires of variable lengths, with external diameters lower than 100 nm with a tubular configuration [25].

**Figure 2:**
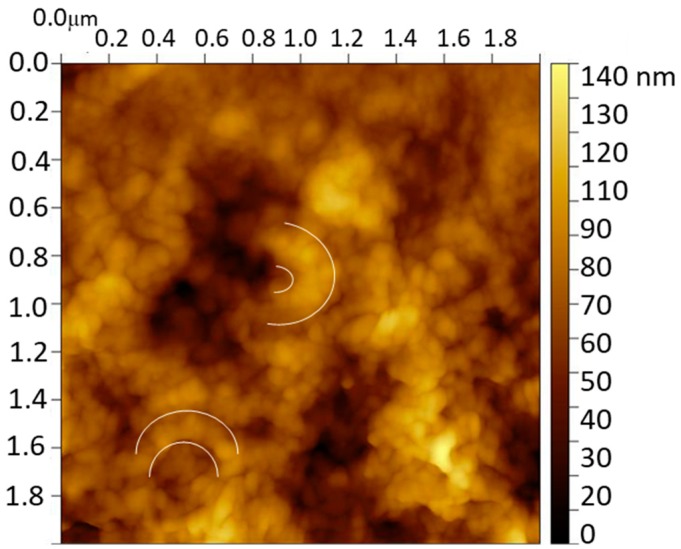
AFM image of the molecularly imprinted CS-MWCNT/AuNP nanocomposite modified BDD electrode. White semicircles show examples of the semicircular globular structures found.

The characterization of the molecularly imprinted CS-MWCNT/AuNP nanocomposite modified BDD electrode was imaged by the AFM technique. Figure 2 shows the image of the MIP sensor after removing the catechol template where the presence of semicircular globular structures with a thickness of 200 nm is remarkable. These structures, repeated over the entire surface, are indicative of the successful CS coating of MWCNT/AuNP. The BDD surface was completely covered with a globular structure of CS film (diameter around 50 nm) that contains the CS coated MWCNT/AuNP circular structures.

### 3.2. Optimization of MWCNT/AuNP Concentration

**Figure 3:**
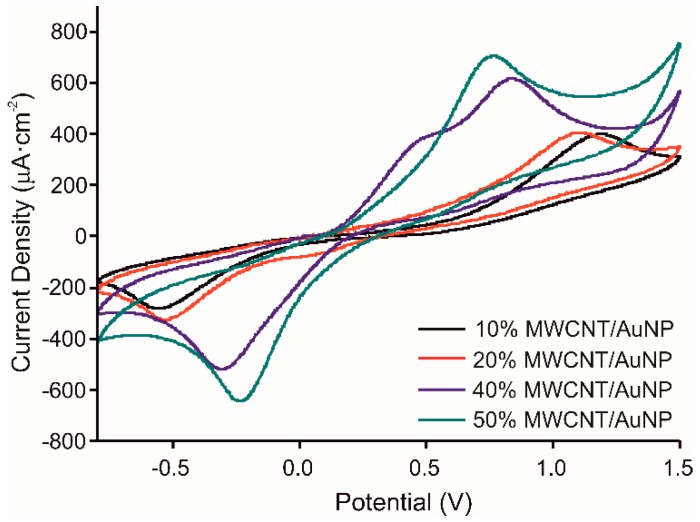
Voltammetric responses of the MIP CS-MWCNT/AuNP nanocomposite modified BDD electrode in 10^−3^ M catechol in 0.1 M, pH 7.4 PBS solution with increased concentrations of MWCNT/AuNP composite, 10% in black, 20% in red, 40% in blue and 50% in green.

The chitosan/catechol ratio was kept similar to our previous work [22]: CS concentration 1.5 mg/mL, catechol concentration 0.1 M. The behavior of the prepared molecularly imprinted CS-MWCNT/AuNP nanocomposite modified BDD electrode has been studied for different MWCNT/AuNP concentrations (10, 20, 40, and 50%) tested in 10^−3^ M catechol in 0.1 M PBS solution, pH 7.4, at a scan rate of 100 mV/s from −800 to 1500 mV. Figure 3 shows that the intensity of the anodic and cathodic peaks, corresponding to the oxidation of catechol and reduction of quinone, increases when the concentration of MWCNT/AuNP increases (Intensities: of the anodic peak: 713.8 µA for 50% MWCNT concentration, 618.8 µA for 40%, 411.1 µA for 20% and 404.9 µA for 10%). Moreover, the ΔEp value between anodic and cathodic peaks decreases when the concentration of MWCNT/AuNP increases, showing higher reversibility. However, for 40% of MWCNT/AuNP, two anodic peaks were observed, which was the key point to select this concentration for further experiments, the peak at 345 mV can be attributed to gold oxidation [27].

### 3.3. Electrochemical Characterization of the MIP Cs-MWCNT/AuNP Nanocomposite/BDD Sensors

**Figure 4:**
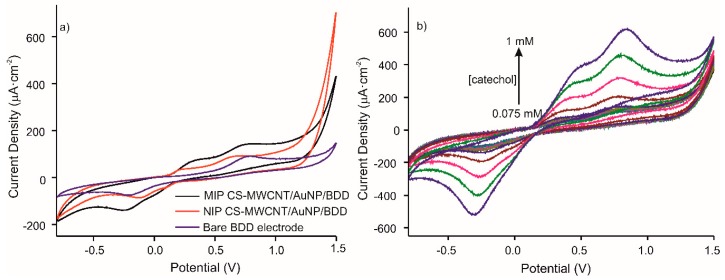
Voltammetric responses of (**a**) MIP CS-MWCNT/AuNP nanocomposite/BDD sensor in black, NIP CS-MWCNT/AuNP nanocomposite/BDD sensor in red and bare electrode in blue, in 10^−4^ M catechol in 0.1 M, pH 7.4 PBS solution and (**b**) MIP CS-MWCNT/AuNP nanocomposite/BDD to increased concentration of catechol (0.075, 0.1, 0.25, 0.5, 0.75, and 1 mM) in 0.1 M, pH 7.4 PBS solution.

The CV response of the optimized MIP CS-MWCNT/AuNP nanocomposite/BDD electrochemical sensor, was studied. Figure 4a shows the voltammetric responses of the MIP and NIP sensors (presence and absence of the template molecule, respectively) and the bare electrode in 10^−4^M catechol in 0.1 M, pH 7.4 PBS solution at a scan rate of 100 mV/s from −800 to 1500 mV. The voltammograms for MIP and NIP, compared to bare gold electrode, demonstrate the presence of the CNT/AuNP composite with the appearance of a second anodic peak produced by the oxidation of gold. The intensity of the anodic peaks, comparing MIP and NIP, increased by a factor of 1.6. The electroactive surface area was extracted from the impedance measurements of the interface. Charge transfer resistance of bare BDD electrode, NIP CS-CNTs/AuNPs BDD electrode, and MIP CS-CNTs/AuNPs BDD after template removal were 461, 8026 and 6992 Ω respectively. Coverage rates of 94% for NIP and 93% for MIP were calculated using following equation: 1-R_ct_(Bare BDD)/R_ct_(NIPs or MIPs/BDD) [28].

Figure 4b represents the responses of MIP CS-MWCNT/AuNP nanocomposite/BDD sensor towards increased concentrations of catechol (0.075, 0.1, 0.25, 0.5, 0.75, and 1 mM). The results show that the intensity of redox peaks increases with catechol concentration for the MIP sensor.

### 3.4. Sensitivity, Reproducibility, Stability, and Selectivity

**Figure 5:**
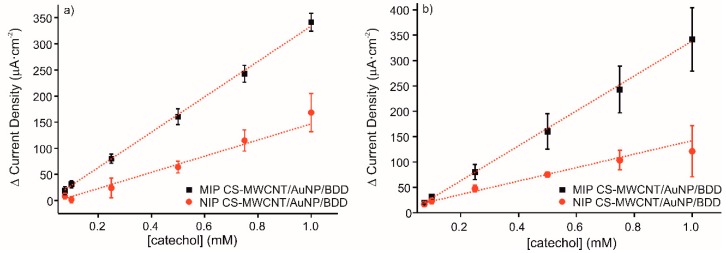
Calibration curves of MIP CS-MWCNT/AuNP nanocomposite/BDD sensor in black and NIP CS-MWCNT/AuNP nanocomposite/BDD sensor in red in 0.1 M, pH 7.4 PBS solution for increased concentration of catechol (0.075, 0.1, 0.25, 0.5, 0.75, and 1 mM) for (**a**) three sensors prepared under the same conditions and (**b**) the same sensor measured three times.

The reproducibility of three different sensors with the same characteristics and elaborated following the same conditions has been measured by cyclic voltammetry. Figure 5a shows the calibration curves for current intensity response, ΔI_anodic,_ to increased concentrations of catechol with the MIP and NIP sensors. The linear equations obtained were y = 339.05x − 4.88 (R^2^ = 0.9985) for MIP CS-MWCNT/AuNP nanocomposite/BDD sensor and y = 154.92x − 8.05 (R^2^ = 0.9521) for NIP CS-MWCNT/AuNP nanocomposite/BDD sensor. The LOD of MIP CS-MWCNT/AuNP nanocomposite/BDD sensor was 3.68 × 10^−5^ M and it was obtained calculating the 3 σ/m criterion, where σ is the standard deviation of three blank signals and m is the slope of the linear calibration curve. The value of LOD obtained with the elaborated sensors indicated an acceptable sensitivity for catechol detection. The ratio of the MIP sensitivity to the NIP sensitivity is equal to 2.2. The RSD for three different sensors was for 4.5% MIP and 12.4% for NIP.

The repeatability of the developed electrochemical sensor to detect catechol was evaluated by cyclic voltammetry. Figure 5b represents the detection of catechol from 0.075 to 1 mM with one electrode reused three times, which was washed prior to each measurement in KCl 0.1 M solution for 20 min in order to remove the phenol adsorbed on the surface. The obtained results demonstrate the excellent performance of the elaborated sensor to recognize the detection molecule with several uses, indicating good durability and robustness. The RSD for three different measures with the same sensor was for 10.8% MIP and 10.2% for NIP.

**Figure 6:**
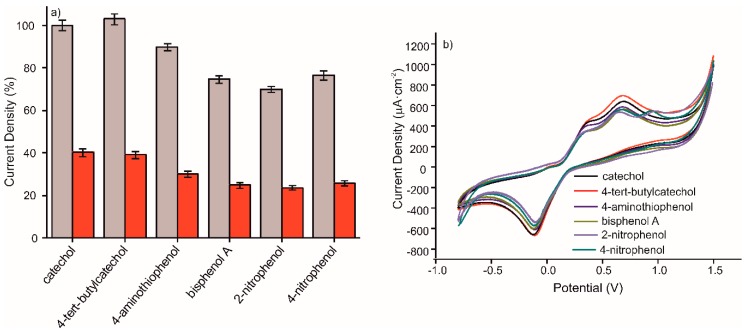
(**a**) Percentage of CV anodic peak value offered by MIP CS-MWCNT/AuNP nanocomposite/BDD sensor in black and NIP CS-MWCNT/AuNP nanocomposite/BDD sensor in red for the detection of catechol 10^−3^ M containing different interfering phenols in 10^−3^ M prepared in 0.1 M, pH 7.4 PBS solution and (**b**) Voltammetric responses of MIP CS-MWCNT/AuNP nanocomposite/BDD sensor for the detection of catechol 10^−3^ M containing catechol (black), 4-tert-butylcatechol (red), 4-aminothiophenol (dark blue), bisphenol A (green), 2-nitrophenol(purple), and 4-nitrophenol (aqua blue) 10^−3^ M prepared in 0.1M, pH 7.4 PBS solution.

The selectivity of the sensor was characterized by the influence of other phenolic compounds, at a concentration of 10^−3^ M, on the anodic peak maximum of catechol at the same concentration. The interference of different phenolic compounds in 10^−3^ M concentration in a 10^−3^ M catechol solution prepared in 0.1M, pH 7.4 PBS solution was critical to test the selectivity of the MIP CS-MWCNT/AuNP nanocomposite/BDD sensor. The sensitivity of detection of the mentioned sensor to phenolic molecules, was compared with that of the NIP CS-MWCNT/AuNP nanocomposite/BDD sensor. Figure 6 shows the percentage for the anodic peak maximum obtained by CV for the detection of 10^−3^ M catechol in the presence of 4-tert-butylcatechol, 4-aminothiophenol, bisphenol A, 2-nitrophenol or 4-nitrophenol. In view of the results, the catechol signal slightly increased in the presence of 4-tert-butylcatechol and decreased by 6% in the presence of 4-aminothiophenol. The effect of phenolic interfering compounds was analyzed in comparison to the RSD. It comes that 4-tert-butylcatechol and 4-aminothiophenol does not have a significant interfering effect whereas bisphenol A, 2-nitrophenol, and 4-nitrophenol are interfering molecules, due to non-specific adsorption. The non-specific adsorption of these compounds prevents the recognition of catechol by the MIP film. The MIP/NIP signal ratio were preserved in presence of these interfering compounds.

The analytical parameters obtained with the MIP CS-MWCNT/AuNP nanocomposite/BDD sensor for phenolic compound detection, such as catechol were compared to previous studies on catechol detection using enzyme-free BDD electrodes, reported in the bibliography (Table 1). The MIP CS-MWCNT/AuNP nanocomposite/BDD sensor presents a rather high detection limit and a very wide dynamic range. This point must be due to a larger number of imprinted sites in this sensor brought about by the inclusion of MWCNT/AuNP.

### 3.5. Detection of Catechol in a Complex Matrix

**Figure 7:**
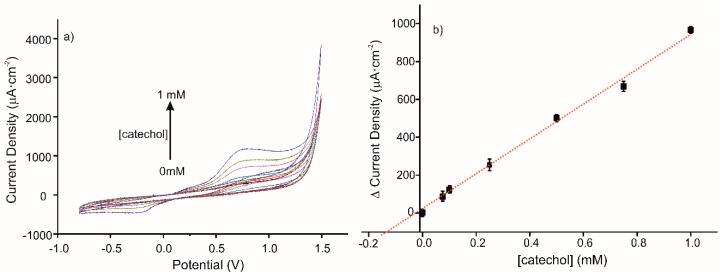
(**a**) Voltammetric responses of MIP CS-MWCNT/AuNP nanocomposite/BDD sensor in 10% diluted wine solution in 0.1 M, pH 7.4 PBS solution, for increased concentration of catechol (0, 0.075, 0.1, 0.25, 0.5, 0.75, and 1 mM). (**b**) Calibration curve for standard additions of catechol in a diluted wine sample (10% diluted in 0.1 M, pH 7.4 PBS solution), using the CS-MIP/BDD sensor.

The detection of catechol in a real sample of red wine with the elaborated sensor was tested by CV. The CV responses to standard additions of catechol (0, 0.075, 0.1, 0.25, 0.5, 0.75, and 1 mM) in the diluted wine sample using MIP sensor are presented in Figure 7a. The corresponding calibration curve is presented in Figure 7b; its equation is y = 919.54 + 23.44 (R^2^ = 0.9936). The ratio of MIP/NIP sensitivities is 1.8, in agreement with the imprinted factor found in a buffer medium. The deduced concentration of catechol in the diluted red wine sample is 170 µM.

## 4. Conclusions

The presented work describes the simple and effective development of a novel, biocompatible electrochemical sensor for the detection of catechol. Electroactive materials such as carbon nanotubes, (CNT) and gold nanoparticles (AuNP) encapsulated in a polymeric matrix of chitosan, Cs were employed in the elaboration of a molecular imprinted polymers (MIP) electrochemical sensor, where a Cs matrix of CNT decorated with AuNP was deposited on the surface of a boron-doped diamond, BDD electrode followed by cyclic voltammetry, CV analysis. The performance as a MIP sensor was excellent, showing perfect affinity to the detection molecule with extraordinary selectivity, with excellent reproducibility, repeatability, and sensitivity. Moreover, the capability of the sensor to measure in complex samples has been tested, obtaining favorable results in the quantification of catechol in red wine samples. For these reasons, this research offers a promising approach to the development of imprinting electrochemical sensors for phenol detection based on biocompatible polymers and sensitive nanomaterials.

## Figures and Tables

**Table 1:** Analytical performance of enzyme-free sensors for catechol detection

Sensor Description	Electrochemical Technique	Sensitivity (µA mM^−1^·cm^−2^)	LOD (M)	Catechol Range (µM)	Ref.
CS-MWCNT/AuNP nanocomposite/BDD sensor	Voltammetry	339.05	3.6 × 10^−5^	75–1000	This work
Electrodeposited CS-MIP/BDD sensor	Voltammetry	3972.00	6.9 × 10^−7^	0–80	[22]
Au/TiO_2_ BDD electrode	Voltammetry	51.58	1.4 × 10^−6^	5–200	[29]
Nanograss array BDD electrode	Amperometry	719.71	6.9 × 10^−^^7^	0–80	[30]

## References

[1] Datta S., Kanjilal B., Sarkar P. (2017). Electrochemical Sensor for Detection of Polyphenols in Tea and Wine with Differential Pulse Voltammetry and Electrochemical Impedance Spectroscopy Utilizing Tyrosinase and Gold Nanoparticles Decorated Biomembrane. J. Electrochem. Soc..

[2] Makhotkina O., Kilmartin P.A. (2010). The use of cyclic voltammetry for wine analysis: Determination of polyphenols and free sulfur dioxide. Anal. Chim. Acta.

[3] Asan A., Isildak I. (2003). Determination of major phenolic compounds in water by reversed-phase liquid chromatography after pre-column derivatization with benzoyl chloride. J. Chromatogr. A.

[4] Stratil P., Klejdus B., Kubáň V. (2006). Determination of totalcontent of phenolic compounds and their antioxidantactivity in vegetables–evaluation of spectrophotometricmethods. J. Agric. Food Chem..

[5] Keshvari F., Bahram M. (2017). Selective, sensitive and reliable colorimetric sensor for catechol detection based on anti-aggregation of unmodified gold nanoparticles utilizing boronic acid–diol reaction: optimization by experimental design methodology. J. Iran. Chem. Soc..

[6] Yue M., Lin Q., Xu J., Jiang T. (2018). Ionic liquid-based headspace in-tube liquid-phase microextraction coupled with CE for sensitive detection of phenols. Electrophoresis.

[7] Ibarra-escutia P., Juarez J., Calas-blanchard C., Louis J., Ramírez-silva M.T. (2010). Amperometric biosensor based on a high resolution photopolymer deposited onto a screen-printed electrode for phenolic compounds monitoring in tea infusions. Talanta.

[8] Rodríguez-méndez M.L., Gay M.D., Saja J.A., Mu R. (2012). Multisensor system based on bisphthalocyanine nanowires for the detection of antioxidants. Electrochim. Acta.

[9] Kilmartin P.A., Zou H., Waterhouse A.L. (2001). A Cyclic Voltammetry Method Suitable for Characterizing Antioxidant Properties of Wine and Wine Phenolics. J. Agric. Food Chem..

[10] Fu Y., Lin Y., Chen T., Wang L. (2012). Study on the polyfurfural film modified glassy carbon electrode and its application in polyphenols determination. J. Electroanal. Chem..

[11] Giuliani E., Fernandes R., Brazaca L.C., Rodríguez-mendez M.L., Saja J.A., Zucolotto V. (2011). Biosensors and Bioelectronics Immobilization of lutetium bisphthalocyanine in nanostructured biomimetic sensors using the LbL technique for phenol detection. Biosens. Bioelectron..

[12] Alessio P., Pavinatto F.J., Oliveira N., De Saja J.A., Constantino C.J.L., Rodriguez-Mendez M.L. (2010). Detection of catechol using mixed Langmuir—Blodgett films of a phospholipid and phthalocyanines as voltammetric sensors. Analyst.

[13] Maikap A., Mukherjee K., Mondal B., Mandal N. (2016). Zinc oxide thin film based nonenzymatic electrochemical sensor for the detection of trace level catechol. RSC Adv..

[14] Ahmad O.S., Bedwell T.S., Esen C., Garcia-cruz A., Piletsky A., Le L. (2018). Molecularly Imprinted Polymers in Electrochemical and Optical Sensors. Trends Biotechnol..

[15] Gopalan A., Komathi S., Muthuchamy N., Lee K., Whitcombe M.J., Dhana L., Sai-anand G. (2019). Progress in Polymer Science Functionalized conjugated polymers for sensing and molecular imprinting applications. Prog. Polym. Sci..

[16] Maciel J.V., Durigon A.M.M., Souza M., Quadrado R.F.N., Fajardo R. (2019). Polysaccharides derived from natural sources applied to the development of chemically modified electrodes for environmental applications: A review. Trends. Environ. Anal..

[17] Zaidi S.A. (2018). Chemical Development of molecular imprinted polymers based strategies for the determination of Dopamine. Sens. Actuators B Chem..

[18] Ou Y., Qin C., Tsen W.C., Wen S., Wang J. (2018). Chitosan—Based composite membranes containing chitosan—Coated carbon nanotubes for polymer electrolyte membranes. Polym. Adv. Technol..

[19] Meng X., Guo W., Qin X., Liu Y., Zhu X., Pei M. (2014). A molecularly imprinted electrochemical sensor based on gold nanoparticles and multiwalled carbon nanotube—Chitosan for the detection of tryptamine. RSC Adv..

[20] Zhou Y., Zhi J. (2006). Development of an amperometric biosensor based on covalent immobilization of tyrosinase on a boron-doped diamond electrode. Electrochem. Commun..

[21] Luong J.H.T., Male B., Glennon J.D. (2009). Boron-doped diamond electrode: synthesis, characterization, functionalization and analytical applications. Analyst..

[22] Salvo-Comino C., Rassas I., Minot S., Bessueille F., Rodriguez-Mendez M.L., Errachid A., Jaffrezic-Renault N. (2020). Voltammetric sensor based on electrodeposited molecularly imprinted chitosan film on BDD electrodes for catechol detection in buffer and in wine samples. Mater. Sci. Eng. C.

[23] Turkevich J., Stevenson P.C., Hillier J. (1951). A study of the nucleation and growth processes in the synthesis of nanoparticles. Discuss. Faraday Soc..

[24] Carpay F.M.A., Cense W.A. (1973). Controlled nucleation for the regulation of the particle size in monodisperse gold suspensions. Nat. Phys. Sci..

[25] David M., Arab M., Martino C., Delmas L., Guinneton F., Gavarri J. (2012). Carbon nanotubes/ceria composite layers deposited on surface acoustic wave devices for gas detection at room temperature. Thin Solid Films.

[26] Li Y., Li Z., Liu H., Chen S., Guo X., Lin M., Li F. (2019). A Portable Electrochemical Platform Integrated with a 3D AuNPs/CNTs Sponge for Point-of-Care Testing of Neurotransmitters. J. Electrochem. Soc..

[27] Rao H., Liu Y., Zhong J., Zhang Z., Zhao X., Liu X., Jiang Y., Zou P., Wang X., Wang Y. (2017). Gold nanoparticle/chitosan@ N, S co-doped multiwalled carbon nanotubes sensor: fabrication, characterization, and electrochemical detection of catechol and nitrite. ACS Sustain. Chem. Eng..

[28] Mlika R., Rouis A., Bonnamour I., Ben Ouada H. (2011). Impedance spectroscopic investigation of the effect of thin azo-calix [4] arene film type on the cation sensitivity of the gold electrodes. Mater. Sci. Eng. C.

[29] Wei M., Liu Y., Gu Z., Liu Z. (2011). Electrochemical Detection of Catechol on Boron-doped Diamond Electrode Modified with Au/TiO_2_ Nanorod Composite. J. Chin. Chem. Soc.-Taip..

[30] Lv M., Wei M., Rong F., Terashima C., Fujishima A., Gu Z. (2010). Electrochemical Detection of Catechol Based on As-Grown and Nanograss Array Boron-Doped Diamond Electrodes. Electroanalysis.

